# Dendritic Cells: A Double-Edged Sword in Immune Responses during Chagas Disease

**DOI:** 10.3389/fmicb.2016.01076

**Published:** 2016-07-14

**Authors:** Natalia Gil-Jaramillo, Flávia N. Motta, Cecília B. F. Favali, Izabela M. D. Bastos, Jaime M. Santana

**Affiliations:** ^1^Laboratório de Interação Patógeno-Hospedeiro, Instituto de Biologia, Universidade de BrasíliaBrasília, Brazil; ^2^Faculdade de Ceilândia, Universidade de BrasíliaBrasília, Brazil; ^3^Laboratório de Biologia do Gene, Instituto de Biologia, Universidade de BrasíliaBrasília, Brazil

**Keywords:** chagas disease, *Trypanosoma cruzi*, dendritic cell, immunoregulation, evasion strategy

## Abstract

Dendritic cells (DCs) are the most important member of the antigen presenting cells group due to their ability to recognize antigen at the infection site and their high specialized antigen internalization capacity. These cells have central role in connecting the innate and adaptive immune responses against *Trypanosoma cruzi*, the causative agent of Chagas disease. These first line defense cells modulate host immune response depending on type, maturation level, cytokine milieu and DC receptor involved in the interactions with *T. cruzi*, influencing the development of the disease clinic forms. Here, we present a review of DCs–*T. cruzi* interactions both in human and murine models, pointing out the parasite ability to manipulate DCs activity for the purpose of evading innate immune response and assuring its own survival and persistence.

## Introduction

More than a century has passed since Carlos Chagas discovered the pathogen *Trypanosoma cruzi* and the natural way of its transmission to humans and animals, and elucidated the corresponding human disease it causes (Kropf and Sá, 2009). Chagas disease is also transmitted through blood transfusions or organ transplants, vertically from mother to child through the placenta, and through contaminated food (Andrade et al., 2014). This neglected disease affects predominantly deprived people and induces social and economic impacts by decreasing patient’s productivity and earning capacity (Hotez et al., 2007).

With 28,000 new cases per year and 8,000 newborns infected during gestation, Chagas disease affects about 8 million people, beyond 65 million live in areas of exposure and are at risk of contracting the disease (World Health Organization, 2015). Despite the efforts to reduce Chagas disease and the number of infections over the last two decades, it is still endemic in Latin America (Rassi et al., 2010; Strasen et al., 2013) and, as result of global migration, the number of people infected with *T. cruzi* is increasing in North America, Europe, Japan and Oceania (non-endemic areas) (Schmunis, 2007). Since Chagas disease crosses boundaries and spreads, it becomes not only a burden for the endemic countries but a worldwide health concern (Andrade et al., 2014).

Treatment of Chagas disease is one of the greatest therapeutic challenges in tropical medicine since the only drugs approved for human treatment - Nifurtimox and Benznidazole - date from the early 1970s and have carcinogenic properties conferred to nitrofuran and nitroimidazole, their active chemical groups, respectively (Bern, 2011; Wilkinson et al., 2011). Nevertheless, both medicines share similar features: necessity of prolonged administration, effectiveness related to the acute phase and different susceptibility among *T. cruzi* strains (Bermudez et al., 2015). Aditionally, Nifurtimox prescription has been abolished in Brazil, Argentina, Uruguay, Chile and US due to its toxic effects over central nervous system, genotoxicity, and reduced efficacy (Wilkinson et al., 2011).

The natural disease progression consists of an acute phase that can be followed by an asymptomatic indeterminate phase, which represents most of the cases and, one-third of infected population progress to the chronic phase that may lead to death (Machado et al., 2013). Early clinical manifestations include headache, fever, and cough, which are non-specific signs; consequently most of infected individuals are neither notified nor treated. Symptomatic chronic stage of the disease usually occurs from 10 to 25 years after infection and typical manifestations are mild to severe cardiomyopathy and/or dilated digestive tract (megaoesophagus and megacolon) (Steverding, 2014). The mechanisms responsible for patient progression from the indeterminate to the symptomatic chronic phase are not completely understood, although the immunological events initiated during the acute phase undoubtedly drive the organism toward the development of a protective or deleterious immune response (Andrade et al., 2014).

It is unanimity that during the acute phase, dendritic cells (DC), along with macrophages, and natural killer (NK) cells, guarantee the host first line of defense against the parasite (Watanabe Costa et al., 2016). Guide by this context, our purpose is to present a review about DC–*T. cruzi* interaction pointing out the parasite ability to evade innate immune response to assure its own survival and persistence.

## Trypanosoma cruzi

*Trypanosoma cruzi* is an obligate intracellular protozoan of the Kinetoplastea Class, characterized by the presence of one flagellum and a single mitochondrion comprising the kinetoplast, a specialized DNA-containing structure. The Order Trypanosomatida comprises other parasites responsible for severe diseases in humans and other species, for instance *Leishmania* sp. (leishmaniasis), *Trypanosoma brucei* (African trypanosomiasis), *Phytomonas* sp. (plant diseases), and *Crithidia* sp. (arthropods diseases) (Stevens, 2008), that show adaptability toward their hosts with numerous sophisticated immune evasion strategies (D’Avila-Levy et al., 2015).

*Trypanosoma cruzi* strains present diversity in morphology, infection capacity, cell surface predominant antigens and other biological and biochemical features (de Lana et al., 2010). In 2009, *the Second Satellite Meeting* held in Buzios, Brazil suggested six Discrete Typing Units (DTUs) for classifying the several *T. cruzi* strains, named TcI to TcVI (Zingales et al., 2009). A DTU is a population set that is more genetically related among themselves than to any other population, showing common genetic, immunological and molecular markers (Tibayrenc, 1998). These DTUs subsets have different distribution among the American continent due to parasite adaptations to different vectors and reservoirs (Zingales et al., 2012), and, although, some characteristics are shared within the same DTU, differences in many aspects can be found among strains. *T. cruzi* Colombian and G strains, belonging to TcI group, are a clear example of this intra-DTU diversity. Colombian strain, isolated from humans, is highly infective (Ramírez et al., 2010), but no human infections were reported from G strain, which was isolated from anal gland secretions of an opossum (Deane et al., 1984). *T.* cruzi G strain has gp35/50 as predominant surface glycoprotein that appears to be related to its poor internalization by humans cells (Yoshida, 2006).

*T. cruzi* life cycle alternates between reduviid bug vectors and mammal hosts. Domestic and wild animals like opossum, bats, armadillo, and monkey may also act as reservoir host (Coura et al., 2002). The parasite presents different forms during its life cycle – epimastigotes remains in the insect gut; non-divinding and infective metacyclic trypomastigotes (MT) find in the insect feces and/or urine; bloodstream trypomastigotes that can circulate in the mammalian blood and, finally, the intracellular, proliferative and rounded amastigote form. A triatomine insect picks up the parasite trypomastigote forms by feeding on the blood of an infected mammal (Teixeira et al., 2009) and, once inside the vector, those forms differentiate into epimastigotes and multiply in midgut (Manchola et al., 2015). After migration to the bug’s hindgut, epimastigotes attach to the waxy gut cuticle by their flagella and differentiate into infectious MT, which will be deposited along with feces/urine on the skin of the victim (Muñoz-Saravia et al., 2012). The parasite penetrates the new host through lesions caused by its bite or a variety of mucosal membranes. MTs invade host cells entering a parasitophorous vacuole (Romano et al., 2012), which fuses to lysosomes. Once free in the cytoplasm, the parasite differentiates into amastigotes that undergo binary fission multiplication and transform back to trypomastigotes, which are released upon rupture of the host cell membrane and infect neighboring cells or enter the bloodstream (Stecconi-Silva et al., 2003; Tonelli et al., 2004). Bloodstream trypomastigotes may begin another infection cycle when they are taken in the blood feeding of the vector (Bern, 2015). Humans and animals can be infected orally through the ingestion of food and drink contaminated by crushed infected insect vectors or their feces (Nóbrega et al., 2009; Bastos et al., 2010).

Immune evasion strategies developed by *T. cruzi* are essential to the establishment of a long-life infection. Complement inactivation, escape from phagolysosome, antibodies with no *T-cruzi* specificity and delayed immune response are some examples of those strategies (Reviewed by Nardy et al., 2015; Cardoso et al., 2016). Another mechanism involved in parasite persistence is the manipulation of DC functions, which impairs an adequate host immune defense.

## Dendritic Cells

Dendritic cells are bone-marrow-derived cells that belong to the antigen presenting cells (APC) group, being considered the most important member due to their high capacity to recognize and internalize antigens at the infection site (Haniffa et al., 2009; Collin et al., 2013). They link innate and adaptive immune responses by capturing, processing and expressing antigens in the cell surface membrane (Planelles et al., 2003; Steinman, 2007). Immature DCs have a wide range of innate receptors that enables the recognition of pathogens via pattern recognition receptors (PRRs), which activate DCs through signaling pathways eliciting their maturation (Pearce and Everts, 2015). Toll-like receptors (TLRs), abundant in APC, are one of the best-characterized PPRs and efficiently detect pathogen-associated molecular patterns (PAMPs) that are located on the cell surface or in the lumen of endosomes. The presence of TLRs on the cell surface or in the lumen of endosomes enables efficient pathogen recognition and the development of an adequate innate immune response; i.e., TLR2, located in DCs plasma membrane, senses various components of pathogens and its stimulation induces the production of various proinflammatory cytokines. TLR9, located in DCs endolysosome, is involved in virus, bacteria, protozoa nucleic acid recognition and its activation also leads to the production of proinflammatory cytokines (Takeuchi and Akira, 2010). After antigen recognition, DCs can travel along the body from peripheral tissues to lymphoid organs/tissues where they present the processed antigens through their major histocompatibility complexes (MHC) to T cells (Lipscomb and Masten, 2002). Maturation process comprises differentiation from antigen-capturing specialized cells to presenting and stimulating specialized cells. Mature DCs can be identified by morphological aspects like cytoplasmic extensions and abundant intracellular structures (lysosomes, endosomes, granules, etc.) related to antigen processing and by modulation of molecular markers such as up-regulation of CD83, of co-stimulatory molecules like CD80 (or B7-1) and CD86 (or B7-2) and MHC (O’Neill et al., 2004).

Dendritic cells present antigens to lymphocytes CD8^+^ and CD4^+^ T by MHC class I and MHC class II, respectively (Blum et al., 2013). For MHC class I molecules, these antigens originate from intracellular sources; for MHC class II, from exogenous sources. Some DCs have an atypical ability, called cross-presentation that allows to load peptides from exogenous antigens onto MHC class I molecules (Vyas et al., 2008; Neefjes et al., 2011; Segura and Amigorena, 2015). MHC class I molecules are expressed by all nucleated cells and their antigen presentation pathway consists in a series of reactions: (1) intracellular proteins are degraded by the proteasome; (2) the peptides are delivered to the endoplasmic reticulum by the transporter associated with antigen processing complex; (3) antigens are loaded onto MHC class I molecules; (4) peptide–MHC class I complexes are transported via the Golgi to cell surface for presentation to CD8^+^ T cells (Vyas et al., 2008; Neefjes et al., 2011). Recently it was demonstrated that infection of HeLa cells with *T. cruzi* Y strain promotes a down-regulation of the immunoproteasome subunits biosynthesis as well as the MHC class I molecule expression, which could be considered a mechanism of parasite persistence inside the cell (Camargo et al., 2014). Unlike MHC class I expression, MHC class II are mainly expressed by APCs such as DCs, macrophages and B cells (Vyas et al., 2008; Neefjes et al., 2011). Extracellular antigens are taken up by APCs and placed into the phagosome. This compartment fuses with lysosomes to form phagolysosomes, where MHC class II molecules interact with the antigens. Peptide-loaded MHC class II molecules are then transported to the cell surface where they engage antigen-specific CD4^+^ T cells (Vyas et al., 2008; Neefjes et al., 2011). Curiously, MHC II molecules are in constant recycle and degradation process in immature DCs, but mature DCs exhibits a stable and prolonged antigen presentation (den Haan et al., 2014). It is worth mentioning that DCs antigen presentation is not enough for T lymphocytes activation and proliferation. Co-stimulatory molecules expression and cytokine production are also required and they are efficiently provided by mature DCs (den Haan et al., 2014). Following activation via TLRs, DCs may produce acute innate cytokines involved in local and systemic responses such as IL-1β, IL-6, IL-8, IL-12, and TNF-α (Verhasselt et al., 1997; Holdsworth and Can, 2015), however, DCs, under specific conditions, are also able to produce IL-10 and TGF-β for directing a regulatory response (**Table 1**).

**Table 1:** Study conditions in murine models.

DC source	Markers	*T. cruzi* strain	Study conditions	Disease phase	Result	Reference
Spleen	CD11c	Tehuantepec	Tehuantepec *in vivo* infection	Acute	↓CD86; ↓ migration capacity	Chaussabel et al., 2003
Bone-marrow	CD11b; CD11c	RA	RA *in vitro* infection	Acute	↓Cytokine production; ↓endocytic capacity	Poncini et al., 2008
Spleen	CD11c	RA	RA *in vivo* infection	Acute	↓Cytokine production; ↓MHC II expression	Alba Soto et al., 2003
Spleen	CD11c	K98	K98 *in vivo* infection	Acute	↑Co-stimulatory molecules expression; ↑MHC II expresion	Alba Soto et al., 2003
Bone-marrow	CD11b; CD11c	AQ1.7, MUTUM (TcI); 1849, 2369 (TcII)	*In vitro* infections	Acute	↑Anti-inflammatory cytokine production; ↑ TLR2 expression	da Costa et al., 2014
Spleen	CD11c	Y	*In vivo* infection using different mouse lineages	Acute	↓Co-stimulatory molecules expression; ↓MHC II expression	Planelles et al., 2003
Bone-marrow	CD11c	Queretaro	*In vivo* infection using MIF-deficient mice	Acute	IL-12 role in protection	Terrazas et al., 2011
Bone-marrow	CD11b; CD11c	RA	IL-10-deficient DCs were injetec along RA strain in mice	From acute to chronic	↓Cytokine production; ↓MHC II expression	Alba Soto et al., 2010
Bone-marrow	CD11b; CD11c	RA	*In vitro* infections	Acute	↑Anti-inflammatory cytokine production; ↓T cell induction	Poncini et al., 2010
Bone-marrow DCs and NK cells	CD11c	RA	Study of NK cells and DCs interaction in mice	Acute	Unbalanced DC population	Batalla et al., 2013
Spleen	CD11b; CD11c	Colombian	300, 3000, and 30000 initial inocula in mice	From acute to chronic	Less favorable host response in medium inocula	Borges et al., 2012
Bone-marrow		CL Brener	*In vitro* assay using CpG islands	Acute	Recognition by TLR9; ↑IL-12 and IFN-γ production	Bartholomeu et al., 2008
Spleen	CD11c	Y	*In vivo* infection studying TLR9 expression	Acute	↑Recognition by TLR9; ↑inflammatory cytokine production	Gravina et al., 2013
Bone-marrow		Tulahuen	*In vivo* and *in vitro* infection using MyD88/TRIF-deficient mice	Acute	No IFN-β production and no parasite clearance	Koga et al., 2006
Spleen	CD11c	CL Brener	*In vivo* infection using UNC93B1deficient mice	Acute	No correct TLR activation; ↓IL-12p40 and IFN-γ	Caetano et al., 2011
Spleen	CD11c	Dm28c	*In vitro* infections using B2R-deficient DCs	Acute	No IL-12 production	Monteiro et al., 2007
Spleen	CD11c	Dm28c	*In vitro* infections using C5a antagonist	Acute	No IL-12p40/70 and IFN-γ production	Schmitz et al., 2014
Spleen	CD11c	RA	*In vitro* infections using Gal-1-deficient DCs	Acute	Regulatory T Cell induction	Poncini et al., 2015
Spleen	CD11c	Tehuantepec	*In vivo* infections studying Gal-3 expression	Acute	↑Gal-3 expression;↓Migration capacity	Vray et al., 2004
Bone-marrow	CD11c	Tulahuen	*In vitro* infections blocking Siglec-E	Acute	↓Inflammatory cytokine production; ↓T cell induction	Erdmann et al., 2009
Bone-marrow	CD11c	Tehuantepec	*In vitro* infections blocking Siglec-E	Acute	Parasite clearance	Erdmann et al., 2009
Spleen	CD11b; CD11c	Y	*In vitro* infectionn using Slamf1-deficient splenic DCs	From acute to chronic	↓IFN-γ production; no intracellular *T. cruzi* replication	Calderón et al., 2012

Different DC subsets and maturation levels can produce distinct kinds of cytokines or co-stimulatory molecules that can lead either to an inflammatory or a regulatory response. Among DC subsets, myeloid (mDC or classical DC), plasmocytoid (pDC), Langerhans cells (LCs), and derived from monocytes (monocyte DC) are some well-known examples. In murines, mDCs are composed of two main groups: resident and migratory, which are further divided into two subsets: Batf3-dependent and IRF4-dependent DCs (Reviewed by Segura, 2016). mDC Batf3-dependent/migratory expresses CD11c, Clec9A, XCR1, CD103, and CD207; the resident one expresses CD11c, Clec9A, XCR1, and CD8. On the other hand, mDC IRF4-dependent/resident expresses CD11c, CD11b, CD172a; the same markers are found in the migratory subsets along with CD206. The major markers for pDC are CD11c, Ly6c, B220 and SiglecH. For monocyte DC, they are CD11c, CD11b, CD64, Fc𝜀RI, CD206, CD14, CD172a, and Ly6c (Reviewed by Segura, 2016). Finally, LCs is a special DC population present in epidermis and other stratified squamous epithelia, such as oral and genital mucous membranes and bronchus. Despite of being associated with these tissues, LCs may differentiate into migratory cells for antigen presentation; their principal markers are CD11c, CD207, EpCAM, and E-cadherin. Human mDCs classical markers are CD1c^+^, Dectin 1 (CLEC 7A), and Dectin 2 (CLEC6A). Regarding pDCs, CD303 (CLEC4C), CD304 (neuropilin), and CD123 (IL-3R) are human usual markers. Monocyte DCs may express CD14, CD209 (DC-SIGN), CD16, and CD1c (Reviewed by Collin et al., 2013). Lewis and Reizis (2012) have proposed the existence of two more kinds of DCs within these subsets: “receptors”, cells more specialized in capturing antigens and producing cytokines and “presenters”, cells that benefit from these cytokines and travel to lymph nodes for presenting the antigens. It represents another level of specialization among DCs, stating their diversity and their capacity to guide polarity, magnitude and specificity of immune responses (Lewis and Reizis, 2012).

Some studies have demonstrated that acute and chronic phases of Chagas disease require different polarizations of immune response: Th1 profile is protective in an acute state and regulatory responses are important in preventing the chronic phase (Andrade et al., 2014; Dutra et al., 2015). For this reason, DCs are a key group of cells in Chagas disease: they could modulate response depending on type, maturation level, cytokine milieu and DC receptor involved, having a fundamental role in the development of the disease clinic forms including the undetermined stage.

## Dendritic Cell-*T. cruzi* Interaction: Murine Models

Studies on acute Chagas disease in humans are limited due to lack of unique symptoms that characterize this disease state. Nonetheless, extending our knowledge about the acute phase is important because immunological events that take place in this stage have a great influence on the possible development of the chronic phase (Andrade et al., 2014). In this context, experimental murine infection may mimic the human disease, giving us a similar view on what happens at the beginning of *T. cruzi* infection.

**FIGURE 1:**
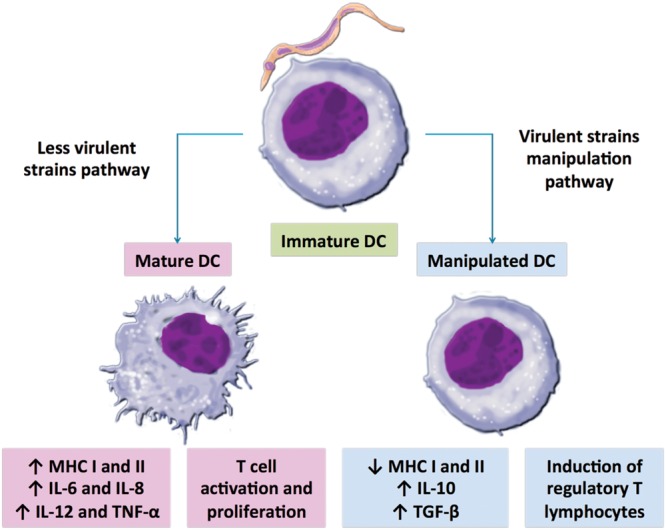
**Possible scenarios after dendritic cells (DCs)-*Trypanosoma cruzi* interaction.** DCs may maturate, up-regulating major histocompatibility complexes (MHC) molecule expression and proinflammatory cytokine production, which may stimulate antigen-specific T lymphocyte activation and proliferation to control parasitemia (magenta); or DCs may be manipulated and the resultant low MHC expression together with high regulatory cytokine production may lead to a weak T cell activation, helping parasite establishment (blue). DC deviant behavior pathway could represent a potent immune evasion strategy of virulent *T. cruzi* strains to successfully set host infection. On the other hand, less virulent *T. cruzi* strains lack DC modulation capacity, which enables host immune response and parasite control.

Unlikely the habitual DCs response during an infection, the expression of important surface molecules like MHC, CD80 or CD86 can be reduced when DCs recognize *T. cruzi* antigens in a strain dependent manner, limiting DC maturation and antigen presenting capacity. During acute phase, splenic DCs infected by *T. cruzi* Tehuantepec strain shows low expression of CD86 molecules and are not able to migrate toward lymphoid organs/tissues (Chaussabel et al., 2003). *T. cruzi* high virulent RA strain (TcVI) induces bone-marrow DCs downregulation of cytokine production and of endocytic capacity added to a suppression of MHC class II, compared to non-infected cells (Poncini et al., 2008). These data are in concordance with the regulation studied by Alba Soto and coworkers, where they also detected a diminished MHC II expression in infected splenic DCs by the same *T. cruzi* strain. Additionally, they showed that the DC manipulated behavior induced by RA strain is not repeated for non-virulent K98 *T. cruzi* strain (TcI) (**Figure 1**; Alba Soto et al., 2003). Another comparative infection study, using mDCs, was performed using TcI (AQ1.7 and MUTUM) and TcII strains (1849 and 2369). The results demonstrated that both *T. cruzi* DTUs may modulate DCs to different extents and this modulation varied more between strains than between DTUs themselves. In general, both DTUs induced the production and expression of anti-inflammatory molecules, such as IL-10, production and PD-L1 and TLR2 expression. Regarding TLR2 expression, it seems that *T. cruzi* has molecules that bind this receptor promoting the production of anti-inflammatory cytokines such as IL-10. Oppositely, proinflammatory molecules did not present a pattern, varying depending on the strain. Finally, they also demonstrated that DC expression of differentiation and activation molecules was not polarized, which suggests that each strain of *T. cruzi* has possibly evolved specific evasion strategies (da Costa et al., 2014). Interestingly, when two mice lineages were infected by high virulent *T. cruzi* Y strain (TcII), the susceptible one showed splenic DCs with reduced capacity of antigen presentation and lower expression of CD40 and CD86 molecules compared with resistant lineages (Planelles et al., 2003). It is well known that the deficiency of co-stimulatory signals during cross-presentation may reduce T cell stimulation or lead to an anergic state (Boussiotis et al., 1996). Thus, it seems that impaired function by DC maybe helps parasite to evade the host immune system.

Cytokine production is another important aspect during DCs-*T. cruzi* interaction, particularly the production of IL-12, considered a protective molecule during the acute infection once it drives a polarized Th1 adaptive response that enables the host to adequately control parasite growth through IFN-γ (Andrade et al., 2014). In concordance, DCs from *T. cruzi*-resistant mouse lineages overexpressed IL-12 and TNF-α while the susceptible lineages produced the Th2 cytokine IL-4 (Planelles et al., 2003). IL-4 is known to mediate host susceptibility to *T. cruzi* but is also required for preventing immune hyperactivity and organ immunopathology (Abrahamsohn and Coffman, 1996; Laucella et al., 1996). Terrazas et al. (2011) demonstrated the protective role of IL-12 through *T. cruzi* Queretaro (TcI) strain infection of MIF (macrophage migration inhibitory factor)-deficient mice. MIF is a pleiotropic cytokine produced by multiple different cell types, including DCs that modulates the expression of several proinflammatory molecules (Cooke et al., 2009). It seems that it favors DC maturation through IL-12 secretion and activation of p38 MAPK protein, a kinase already known to be involved in DC maturation. On the other hand, MIF-deficient mice showed lower levels of IL-12 production and immature bone-marrow DCs, leading to a susceptibility of *T. cruzi* Queretaro (TcI) strain infection (Terrazas et al., 2011). The regulatory IL-10 cytokine is also associated with host susceptibility during acute stage of Chagas disease by limiting DCs induction of antimicrobial effector mechanism such as suppressing DC trafficking to draining lymph nodes (Corinti et al., 2001; Demangel et al., 2002; Alba Soto et al., 2010). A single intravenous injection of IL-10-deficient DCs that were pulsed with parasite antigens conferred an effective control against a lethal challenge with *T. cruzi* RA strain in mice. IL-10 deficient DCs were high Th1 cytokines producers and inducers of antigen-specific T lymphocytes after immunization (Alba Soto et al., 2010). On the other hand, Poncini et al. (2010) have showed that interaction of DCs with *T. cruzi* trypomastigotes was not able to activate the DCs, and these cells became TGF-β and IL-10 producers and were not efficient as lymphocyte stimulators, being classified as regulatory DCs. Furthermore, mature DCs have the capacity to induce NK cells activation and proliferation. NK cells play a significant role in innate immune response and surveillance as a result of their cytokine production and cytolysis of infected cells. Moreover, NK cells secrete IFN-γ, TNF-α, and GM-CSF, which promote DC maturation and activation of T-cell (Gerosa et al., 2002; Piccioli et al., 2002). Batalla et al. (2013) used RA and K98 *T. cruzi* strains to demonstrate the role of NK cells in regulating the maturation level of DCs. During both infections, NK cell was functionally activated and produced IFN-γ but also IL-10; NK cells from mice infected with *T. cruzi* RA strain (high virulence) exhibit reduced ability to lyse and fail to induce maturation of bone marrow-derived immature DCs. This unbalanced DC population could difficult T cell stimulation for an adequate response. Finally, that IL-10 production observed by NK cells after infection with *T. cruzi* RA strain might lead to parasite persistence but can also limit the induction of a vigorous tissue-damaging T-cell response (Batalla et al., 2013). Cytokine production may also be modulated by parasite initial inocula. Three different inocula of *T. cruzi* Colombian strain were used to infect mice, resulting in differential expression of IFN-γ, IL-17, TNF-α, IL-4, and IL-23 by immune cells in heart infiltrates. Curiously, the medium inoculum showed the less favorable host response, which may indicate the existence of an ideal initial inoculum to help parasite evade host immune response (Borges et al., 2012).

It also has been shown the important role of TLRs in *T. cruzi* recognition by first line of defense cells, including DCs. Campos et al. (2004) demonstrated that *T. cruzi* employs a myeloid differentiation factor 88 (MyD88)-, a key adaptor for most TLRs, dependent pathway to elicit cytokine production by the host cells. Intraperitoneal macrophages lacking MyD88 produced less IFN-γ, IL-12, TNF-α and reactive nitrogen intermediates, they also presented higher parasitemia and mortality (Campos et al., 2004). The role of TLRs in the establishment of critical effector mechanisms mediated by CD8^+^ T cells during *T. cruzi* infection was also investigated (Oliveira et al., 2010). The analysis of induction of IFN-γ and cytotoxic activity *in vivo* in TLR2-, TLR4-, TLR9-, or MyD88-deficient mice during infection showed that neither the absence of TLR2, TLR4, or TLR9 individually, nor the ablation of all MyD88-mediated pathways affect the development of cytotoxic and IFN-γ-producing CD8^+^ T cells. Nonetheless, TLR4 deficient macrophages presented a reduced production of TNF-α and nitric oxide (NO), pointing to an important role of the TLR4 pathway and NO production to the innate immune response against *T. cruzi* infection (Oliveira et al., 2010). With regard specifically to DCs, infection with *T. cruzi* parasites promotes recruitment of TLR9 to the DC endolysosome compartment, promoting their interaction during initial phagocytosis. Stimulatory motifs containing CpG islands of *T. cruzi* CL Brener, particularly those formed by genes coding for mucin like proteins, also led TLR9 into lysosomes of bone-marrow DCs and the induction of IL-12 as well as IFN-γ synthesis (Bartholomeu et al., 2008). Such potent proinflammatory activity and, consequently, control of parasite replication could lead to host resistance to infection or avoiding host lethality and maintenance of parasite life cycle long-term parasite persistence. The second hypothesis suggests another adaptation of *T. cruzi* to the host cell-mediated immunity (Bartholomeu et al., 2008). Gravina et al. (2013) stated that DC population constitutes the main source of IL-12/IL-23p40 production in a TLR9-dependent manner in *T. cruzi* Y strain infection. Moreover, when DCs were unable to produce IL-12/IL-23p40, macrophages recovered their capacity to respond to TLR9 agonist, which may represent a compensatory response. Therefore, modulation of TLR9 is important to control the inflammatory response in the different cell populations but TLR9 acts fundamentally on DC inflammatory activity in *T. cruzi* infection (Gravina et al., 2013). Synergy among TLRs in parasite infected DC has also been studied. When MyD88/TRIF (Toll/IL-1R domain-containing adaptor-inducing IFN-β) deficient mice (i.e., with no functional activation of TLRs) were infected with *T. cruzi* Tulahuen strain (TcVI), parasite clearance was impaired mainly by absence of IFN-β production (Koga et al., 2006). In the same work, it was proposed that proinflammatory cytokine production is a MyD88-dependent induction and the expression of IFN-β is a TRIF-dependent. In any case, both TLR adaptors contribute to innate immune responses against *T. cruzi* infection (Koga et al., 2006). UNC93B1, a protein that interacts with TLR3, TLR7, and TLR9, seems to play an essential role in host protection against *T. cruzi* infection (Caetano et al., 2011). UNC93B1 mice deficient were more susceptible to *T. cruzi* infection and produced lower concentration of IL-12p40 and IFN-γ. Such susceptibility was also achieved during TLR3/TLR7/TLR9-deficient mice *T. cruzi* infection, showing that nucleic acid-sensing TLRs are critical determinants of host resistance to primary infection with *T. cruzi* (Caetano et al., 2011).

Other receptors also have been proposed to play an important role during the acute phase of Chagas disease. G-protein-coupled bradykinin (BK) B2 receptors (B_2_R)-deficient mice were more susceptible to *T. cruzi* Dm28c strain (TcI) infection than WT animals (Monteiro et al., 2007). B_2_Rs recognize *T. cruzi* released kinins, mediators related to bradykinin that activate immature DCs (Monteiro et al., 2006). Splenic DCs without B_2_R receptor do not produce IL-12, appointing a critical role for the kinin signaling pathway in the development of type-1 effector T cells (Monteiro et al., 2007). In a recent study, the same group demonstrated that blockage of B_2_R along with C5a receptor resulted in splenic DCs unable to produce IL-12p40/70 and IFN-γ (Schmitz et al., 2014). C5a is an anaphylatoxin derived from proteolysis of C5 complex of complement system, whose biological function is to activate cells from myeloid lineage (Klos et al., 2009). Yet, they showed that, as for kinins, C5a molecules seems to be produced through *T. cruzi* cruzipain activity during infection and can promote DC activation and a Th1 protective response (Schmitz et al., 2014). Galectins, a lectin receptor, can also act as pathogen recognition receptors and as modulators of innate and adaptive response (Vasta, 2009). It has been shown that those receptors are widely expressed in B cell, macrophages and DCs during *T. cruzi* infection (Vray et al., 2004; Zúñiga et al., 2001a,b). Concerning DCs, Galectin-1 seems to be a negative immune regulator that limits the host protective response by driving tolerogenic circuits in DCs. Those tolerogenic DCs induce regulatory T cells activation, which would favor parasite persistence in host tissues or limit collateral tissue damage through suppression of inflammatory responses (Poncini et al., 2015). Galectin-3 (Gal-3) and its specific ligands were over-expressed in splenic DCs after infection by *T. cruzi* Tehuantepec strain, which lead to DC increased adhesiveness and reduced migration (Vray et al., 2004). Therefore, *T. cruzi* modulates Gal-3 and its ligands functionality to improve infection, another immunomodulatory property of *T. cruzi* (Vray et al., 2004). Another lectin-like receptor expressed by immune system cells, Siglec-E (sialic acid-binding Ig-like lectin-E), has also been implicated in *T. cruzi* infection. It is well known that transference of sialic acid by *T. cruzi trans*-sialidase (TS) from host cell to parasite surface mucin-like molecules confers resistance to human complement, contributing to infection (Tomlinson et al., 1994). In this context, pathogenic *T. cruzi* Tulahuen strain (high TS activity) interacted more to Siglec-E than non-pathogenic *T. cruzi* Tehuantepec strain (reduced TS activity). This interaction led to an inhibitory effect on DCs modulation, suppressing the production of cytokine IL-12 and subsequent T-cell activation. In contrast, *T. cruzi* Tehuantepec strain could not install an important parasitemia (Erdmann et al., 2009). Together, those findings suggest that *T. cruzi* (or parasite products) may lead to immunosuppression through its interaction with DC lectin receptors (Terrazas et al., 2010). Slamf1 (self-ligand adhesion molecule - CD150) is a co-stimulatory molecule present in myeloid lineage and required at the interface of antigen presenting cells and T cells (van Driel et al., 2016). *In vitro* and *in vivo* experiments revealed that Slamf1-deficient myeloid cells showed altered production of cytokines and reduced parasite replication. For instance, much lower IFN-γ production was detected in the heart of Slamf1 deficient mice than in the heart of WT mice. Additionally, Slamf1 deficient mice presented reduced cardiac damage despite of the comparable number of infiltrating DCs, macrophages, CD4 and CD8 T lymphocytes to that of WT animals. Therefore, *T. cruzi* requires Slamf1 to replicate in DCs and its absence leads to less production of myeloid cell specific factors by DCs, which are key compounds to host immune response and infection outcomes (Calderón et al., 2012).

Currently, there is an enormous amount of data that states a direct association between mouse DC functional specializations (antigen presentation or pathogen sensing) and their subsets. However, after our meticulous review of literature in the field, it is not unrealistic to conclude that the role of DC subsets in innate immune response against *T. cruzi* needs to be more properly addressed by researches since most cited works emphasize only one surface phenotype as if all DCs are equally functional. The same statement is valid for *T. cruzi* strains, a missing pattern respect to DCs subsets and their level of activation and to *T. cruzi* strains, which hampers an association concerning published data. **Table 1** summarizes the limited actual data in the murine DC- *T. cruzi* system.

## Human Dc-*T. cruzi* Interaction: *In Vitro* Studies

The first experiment that confirmed the ability of *T. cruzi* to infect and reproduce inside a human DC was performed in 1999 (Van Overtvelt et al., 1999), a biological process that had already been known for *Leishmania* (Moll et al., 1995). The authors also demonstrated that DCs, derived from monocytes and infected with *T. cruzi* Tehuantepec strain, significantly reduced HLA-DR and CD40 expression. In addition, these infected DCs were neither IL-12 nor TNF-α producers (Van Overtvelt et al., 1999). In a different study, using the same *T. cruzi* strain, Van Overtvelt and co-workers showed that *T. cruzi* soluble factor(s) released by the parasite itself into the DC culture medium inhibits LPS induced MHC class I up-regulation on the surface of human DC. Such inhibition may decrease the protective effect of specific CD8^+^ T since infected DCs had a weaker capacity of cross-presentation. This reduction of DC function may influence the *in vivo* host’s ability to competently combat *T. cruzi* infection (Van Overtvelt et al., 2002). It is well known that a small family of type 1 glycoinositolphospholipids (GIPLs) is abundant in *T. cruzi* cell surface and, therefore, such molecules seem to have immunoregulatory activities (Brodskyn et al., 2002; Medeiros et al., 2007). GIPLs isolated from *T. cruzi* G (TcI) and Y (TcII) strains were incubated along with LPS to stimulate DCs derived from monocytes. The results showed that *T. cruzi* GIPL antigens direct the down-regulation of both proinflammatory cytokines, such as TNF-α and IL-12 and anti-inflammatory, such IL-10, in DCs. The parasite GIPL also inhibited the expression of co-stimulatory molecules HLA-DR, CD83, CD86, CD80, and CD40 on DC surface. Similar results were achieved when the ceramide portion of GIPL molecule alone was used to stimulate DC, suggesting that fragments from the parasite glycoproteins could represent an evasion strategy of *T. cruzi*. Altogether, GIPL seems to contribute to parasite protection from the innate responses, allowing the beginning of infection and also acts in an inhibitory way on DC maturation, postponing an adequate immune response against *T. cruzi* (Brodskyn et al., 2002). Otherwise, a parasite released protein belonging to thiol-disulfide oxidoreductase family (Tc52) binds to and induces human and murine DC maturation by TLR2 activation. DCs derived from monocytes treated with Tc52 showed higher expression of CD83, CD86, CD54 and HLA-DR and an elevated production of IL-8, MCP-1, MIP-1α. These *in vitro* data suggest that Tc52 may provide local recruitment and activation of leukocyte and then DC migration to the lymph node, where they can trigger B and T cell immune responses (Ouaissi et al., 2002).

**FIGURE 2:**
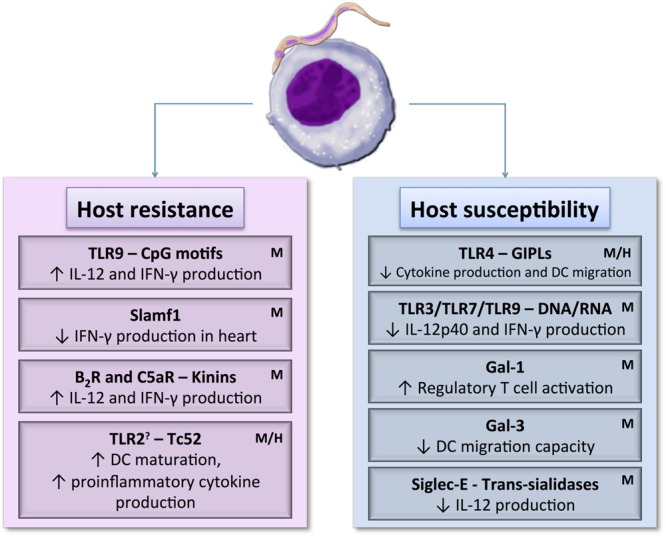
**Dendritic cell receptor–*T. cruzi* antigen interactions may induce host resistance or susceptibility to parasite by activating different DCs signaling pathways.** DCs maturation, cytokine production, and migration capacity could be up or down-regulated depending on the signaling pathways triggered during the initial contact between DCs and the parasite. **H**: DC receptors already known to interact with parasite in human model. **M**: DC receptors already known to interact with parasite in murine model. **?**: May act as a maturation inducer or as regulatory cytokines activator.

Yet, *T. cruzi* Tulahuen strain parasites enhance expression of CD40 and CD80 on cord blood mDCs in a higher level compared to mDCs from adult donors. CD8^+^ T cells proliferation was also stimulated by those cord blood mDCs. In early life, immune responses are considered of partial effectiveness, owing to the relative immaturity of the human immune system therefore it is possible that maternally transmitted IgGs might contribute to overcome some deficiency of fetal/neonatal DCs and to protect the fetus/newborn against pathogens that have already come into contact with the mother (Rodriguez et al., 2012a). Another study, performed by the same group, showed that *T. cruzi* can induce maturation of this DC type without infecting them. Rodriguez and coworkers found that blood cord and adult mDCs that had contact with parasite but were not yet infected also expressed high levels of CD80 and CD83. In addition, they demonstrated that either infected mDCs or *T. cruzi* lysates co-incubated mDCs have a similar expression pattern of their surface molecules. The authors also showed that infection rate in mDCs is lower than in monocytes and granulocytes, maybe due to their enhanced capacity of phagocytosis when compared to mature DCs (Rodriguez et al., 2012b). Consistent with the results shown in murine model, modulation of DCs function varies according to *T. cruzi* strain. In a comparative study, Magalhães and colleagues demonstrated that *T. cruzi* Col cl1.7 (TcI) but not Y (TcII) strain induces higher CD80 and CD86 expression, while *T. cruzi* Y strain induces up-regulation of IL-10, TNF-α and granzyme A production. Also, CD8^+^ T lymphocytes activated by Col cl1.7 strain produced higher level of IL-17. Then, TcI strain were capable of a higher monocyte activation, while the profile induced by TcII was more inflammatory (Magalhães et al., 2015). **Figure 2** summarizes DC receptors referred to in this review.

The present knowledge about the interaction between human DCs and *T. cruzi* is restricted to *in vitro* models where expression of some cytokines and surface molecules were analyzed, but the specific functions of human DC subsets are only beginning to be unraveled. Contradictory studies have been published concerning DC–*T. cruzi* interactions, both in human and mouse models. However it seems that *T. cruzi* virulent strains probably take advantage of susceptible DCs to overcome host immune response and successfully install the infection. Remarkably, those modulated DCs will conduct a weak adaptive response with low expression of MHC class I and II molecules and proinflammatory cytokines, which are fundamental for controlling parasite survival (Poncini et al., 2008, 2010; Alba Soto et al., 2010; da Costa et al., 2014).

## Future: Needs and Expectations

Dendritic cells are crucial decision-making cells of the immunological system as they direct tolerance, anergy, and initiation/regulation of the adaptive immune responses (Banchereau and Steinman, 1998). For these reasons, DC has been proposed as targets for immunotherapy in diseases related to autoimmunity and exacerbated immune responses, such as autoimmune encephalomyelitis (Menges et al., 2002), thyroiditis (Verginis et al., 2005), and arthritis (Jaen et al., 2009) or to improve unsatisfactory immune responses toward tumor or pathogens (Dubsky et al., 2005). Targeting these cells with recombinant antibodies conjugated to autoantigens or pathogen antigens could direct a less exacerbated response against certain disease (Lutz, 2012).

ASP-2, an amastigote protein from *T. cruzi* Y strain, was conjugated with αDEC205 antibody, DEC205 is a C-type lectin endocytic receptor expressed in some DC populations and is widely used for targeting DCs. Recombinant ASP-2/DEC205 antibody was injected in mice, resulting in higher IFN-γ production by splenocytes and high proliferation of antigen-specific CD4^+^ T cells (Rampazo et al., 2015). Thus, targeted regulatory DCs could be used as an immunotherapy strategy during disease undetermined form to prevent evolution to the chronic phase. Nevertheless, host receptor and parasite antigen should be carefully elected because they could influence maturation signals received by DCs for orchestration of the immune response (Cohn and Delamarre, 2014). Furthermore, trypomastigote lysate -pulsed IL-10-deficient DC conferred protection against *T. cruzi* infection to recipient mice by secreting increased amounts of IFN-γ, enhancing antigen-specific production and inducing endogenous DC activation. This DC-based vaccination against *T. cruzi* also demonstrated that IL-10 produced by sensitizing DC has a key role in inhibiting the protection response (Alba Soto et al., 2010). DC-based vaccine has also been successfully tested in *Leishmania*. Freshly isolated pDC from mice pulsed with *Leishmania* antigen and reinjected into host resulted in a protective effect, presenting mixed Th1/Th2 response with secretion of IFN-γ, IL-4 and IL-10 (Remer et al., 2007).

Unfortunately, DC immunizations have an elevated cost, which turns up this approach less attractive added to relative efficiency of the current treatment for Chagas disease during acute phase (Rassi et al., 2010; Cohn and Delamarre, 2014). Nonetheless, this neglected disease remains with no vaccines or antiparasitic drugs proven efficient in chronically infected adults, when most patients are diagnosed. Thus, future DCs immunization researches could be directed toward treatment for the undetermined stage targeting tolerogenic DCs.

## Conclusion

Although efforts have been devoted to deciphering DCs*-T. cruzi* interaction, there is still much to be investigated before the complete understanding of DC role in the induction of immunity against *T. cruzi*. Moreover, our knowledge about that interaction is mostly based on the regulation, differentiation and function of the DC lineage from mouse. A challenge that needs to be overcome is the difficulty in isolating subsets of DCs from human tissue; only then we might be able to improve the understanding of human DCs in a molecular level and perhaps develop vaccines for the prevention or treatment of Chagas disease. Our review reiterates that *T. cruzi* capacity to modulate host DCs is an indispensable strategy to escape from innate immune response with the purpose of its own survival. Nevertheless, DCs present an efficient machinery to capture, process, and present antigens to T cells and to activate B cells therefore their immunotherapeutic potential may not be disregarded.

## Author Contributions

NG-J: participated in design and manuscript writing. FM: participated in design and manuscript writing. CF: participated in design and manuscript writing. IB: participated in design, coordination, and manuscript writing. JS: participated in design, coordination, and manuscript writing.

## Conflict of Interest Statement

The authors declare that the research was conducted in the absence of any commercial or financial relationships that could be construed as a potential conflict of interest.
